# Medical News and Miscellany

**Published:** 1896-11

**Authors:** 


### Medical News and Miscellany.

**Dr. Vard H. Hulen** has been appointed surgeon to the Eye, Ear, Nose and Throat Department of St. Mary's Hospital at Galveston.

**Dr. C. T. Cooper,** of Taylor, Texas, has, on account of the condition of Mrs. Cooper's health, removed to Alpine, Brewster county, Texas.

---

**Dr. J. L. Cunningham,** of Fort Worth, died at that place on the 18th of October, ult. Dr. Cunningham formerly lived at Hempstead, and also at Austin.

---

**The reasons given** for substituting electrocution for hanging are, that the victim will dy'n a mo' decent manner; and it is less re-volt-ing to spectators.

---

**Dr. G. H. Powell,** of Toomevara, Ireland, was eating an apple, when a wasp, which was in the core, stung him on the tongue, and he died in three hours from the glossitis thus induced.

---

**The Transactions.**—We are authorized to State that Von Boeckmann having printed a few more copies of the Transactions than the contract called for will be pleased to dispose of them at small cost.

---

**Dr. W. C. Thompson** has been appointed by the faculty of the Medical Department Fort Worth University as clinical assistant to Dr. Frank Gray, professor diseases of eye, ear, nose and throat.—*S. W. M. and S. Rep.*

---

**Change of Name.**—The editors of *Mathews' Medical Querterly* announce that with the January issue of that publication its name will be changed to "*Mathews' Quarterly Journal of Rectal and Gastro-Intestinal Diseases.*"

---

**Dr. Stephen Smith** says that "natural talent is necessary in order to study medicine."

Not down this way: here, anybody can "study medicine," but it ought to and does require talent to practice medicine properly.

---

**One of our** pleasant callers during the past week was Dr. H. L. Mann, representing the J. Elwood Lee Co., of Conschohocken, Pa. Dr. Mann has been traveling in Texas for a number of years, and is very popular with the medical profession of this State.

**Our subscribers** who are in arrears will please bear in mind that it takes money to issue a journal; and when they make collections divide with us at least. A remittance *on account* will be thankfully received and taken as evidence of good intentions. All bills were mailed October 1st.

---

**Dr. R. H. L. Bibb,** Chief Surgeon of the Mexican National railroad has been appointed English (speaking) Secretary of the Section of Military, Naval and Railroad Surgery, one of the fifteen sections into which the Pan-American Medical Congress is divided; an excellent selection for the place.

---

**For Sale.**—The JOURNAL offers for sale the surgical instruments of the late distinguished Texas Surgeon Dr. Geo. Cupples. They are in perfect order, having been kept with the scrupulous care for which the doctor was noted, and will be sold singly or altogether, at a greatly reduced price. For particulars write to the JOURNAL.

---

**The Hypnotic Magazine** is the name of the latest journal we have seen. Vol. 1, No. 2, September, 1896. It is "devoted to an investigation of the Science of Hypnotism; its uses and abuses; and its therapeutic possibilities." It is edited by Sydney Flower, and is published in Chicago by the Psychic Publishing Co. Subscription $1.00.

---

**The new** brick building of the Medical Department of the Fort Worth University has been completed, the laboratories all fitted up, and by the time this issue of the *Southwestern Medical and Surgical Reporter* is in the hands of its readers one hundred and fifty or more students will be listening to the lectures of the various professors.—*S. W. M. and S. Rep.*

---

**Dr. Frank D. Boyd,** of the editorial staff of the *Southwestern Medical and Surgical Reporter*, has been appointed assistant to the chair of physiology, Medical Department Fort Worth University, with title of lecturer on physiology. This appointment was made by the unanimous vote of the faculty at the request of the professor of physiology.—*S. W. M. and S. Rep.*

---

**Married,** at Cleburne, Texas, September 29, 1896, Dr. Eugene Bryce Osborn to Miss Maude Imogene Richardson, all of

Cleburne. Dr. Osborn is a son of Dr. Jas. D. Osborn, and grandson of Dr. T. C. Osborn, of Cleburne, and is a graduate of the Medical Department University of Texas, class of 1894. The JOURNAL extends its congratulations.

---

**Married,** at Galveston, October 21 (ult.), Dr. W. Beverly West, of Fort Worth, to Miss Alice Mensing, of Galveston. The JOURNAL acknowledges the courtesy of cards, and extends hearty congratulations to its handsome young friend. [Dr. West is Professor of Diseases of Children in the Medical Department of the Fort Worth University.—ED.]

---

**In Chicago,** or, rather—on Lake Michigan—they have a black cat farm, and raise black cats for the fur. In Arizona a mountaineer is making a fortune on rattlesnakes. He derives a big income from the oil, which he sells to druggists, it being regarded in that section as a specific for almost everything; the skins he sells to cowboys for hat bands; while the skeletons he mounts and sells to museums and colleges.—*Scientific American.*

---

**Scabies.**—Dr. Hare, in *Medical World*, says: "The 'itch' (scabies) is often hard to treat successfully. Sulphur ointment, well rubbed in, will often allay, but frequently fails of curing because of the depth of the furrows made by the female acarus. It is therefore best, before the application of the ointment, to give the patient a thorough hot bath, lasting half an hour, with strongly alkaline soap, in order to soften the epidermis and uncover the burrow of the worm. The ointment may then be used with much benefit."—*N. Y. Med. Record.*

---

**Death of Prof. Erichson.**—Sir John Erich Erichson, the great English surgeon,—the father of the "railroad spine," died at his home on 23rd of September, 1896. He visited the United States in 1873, and is well remembered by those who had the honor of meeting him. He was a typical gentlemen, courteous in manner, and upright and just in all his dealings—a general favorite. Dr. Erichson was a very old man and though long since retired from active practice, he continued to be a conspicuous figure in profession circles to the day of his death. His Science and Art of Surgery is his imperishable monument.

**Dr. W. B. Brooks,** of Dallas, editor and owner of the Texas *Courier-Record of Medicine.* died in that city, October 4th, ult.

Dr. Brooks was born in Smith county, Texas, and was about fifty-six years of age. He graduated at the Medical College of St. Louis; class of 1876. He was twice married; his second wife, who survives him, being a Miss Wilson of Fort Worth. Dr. Brooks' oldest son, Odin Brooks, is studying medicine; has taken one course in lectures, and will, with the aid of the younger brother, continue the publication of the *Courier-Record of Medicine.* The JOURNAL extends its condolence to the family.

---

**If there are any** who doubt the necessity of a good law on the subject of practice of medicine, let them read the "Song of the Peripatetic" in this issue. It is not an overdrawn picture, by any means.

Let every Texas physician consider himself a committee of one on the subject of medical legislation; and make it a point to thoroughly explain the situation to the representative from his county, and thus make him see the necessity of *restricting* the *privilege of practicing medicine* to those who can give to a legally-constituted authority satisfactory evidence of the proper qualifications; it matters not how, when or where he "qualified."

---

**An Astringent and Antiseptic Injection for Uterine Leucorrhœa.**—The *Gazette hebdimadaire de medecine et de chirurgie* for August 20th, publishes the following formula, which is attributed to M. A. Bustillo Lirola:

℞ Tannic acid..............................900 grains
Pure alcohol, Creosote, } each..................450 "
Distilled water.......................... 7.5 ounces.
M.

From three to four injections a day are to be taken, for each of which a dessertspoonful of the solution is to be diluted with a pint of warm water.

---

**"A Tasteless Medicine Cup"** is advertised. It consists of a glass tumbler with a partition of glass reaching from the bottom to nearly the top; and a hard rubber septum that overlaps the glass one at top and reaches to nearly the bottom, leaving a space between them. The disagreeable medicine—in liquid form is put in one side and some wine or flavored liquid of some kind is put in the other side. The principle is that the wine or other destroyer of taste "rinses the mouth before the patient ceases to swallow in the act of taking the medicine." If this is a success it is a desideration, surely.

---

**A Doctor Indicted for Murder.**—A Dr. D. E. Thrash, at Houston, said to be a greybeard, was indicted for the murder of a fifteen year old girl named Lily Jarvis, mention of which was made in the associated press dispatches some time ago, and the case written up in the Houston *Post*, all but this doctor's name. The death of the girl was the result of an operation for abortion, we understand. On the 22d of October the "doctor" was admitted to bail, $3000; and his trial set for the 28th of last month. By the time this is read the case will have been disposed of, and if guilty, the "doctor" will spend the remnant of his worthless life where he belongs, having been put where he can do most good for the State. God grant that every abortionist may be caught up with and given his deserts. We have tried to find out something about this doctor, but he seems to be a new comer in Houston, from nowhere in particular.

---

**A Sad Death.**—Lena May, daughter of Dr. and Mrs. W. L. York, and wife of Dr. R. L. Miller was born December 26, 1875, and fell asleep in Jesus at her father's home in Decatur, Texas, September 29, 1896.

This lovely woman, in the bloom of life, a universal favorite of friends, young and old, a devout Christian, an only child of fond parents, the beloved wife of Dr. R. L. Miller, was stricken down just at that time when her loss would be most widely and deeply felt. She received every advantage that parents and grandmother could provide, shielding her from the burdens and hardships of life, and looking to her moral and intellectual culture. What is it to watch the unfolding of the physical and intellectual powers of a dear child, and just when filled with many high hopes and pleasing anticipations concerning its future, to have the dear object of our love taken from affection's embrace? What is it for the husband and her to have had a union of hearts from childhood, and just when their fond hopes were realized, a neat little home fitted up, that this sweetest of earthly ties should be severed in three short months? Her funeral services were conducted in the same room where she was led as a bride on the 24th of June, arrayed in the same dress for both occasions, indeed, "adorned as a bride to meet the bridegroom." How singularly mixed are human joys and sorrows! May He who "loveth whom He chasteneth," sanctify this sore bereavement to all who are exercised thereby, and soothe their aching hearts.

"A life so blameless and so pure are hers,
Leaves only sweetest memories to cheer
The desolate hearts of those who mourn and prove
A bright example while they linger here,
As a clear star that shineth through the night,
To lead them on mid devious ways to everlasting light."

C. M. D. in *Decatur News*.

The JOURNAL extends its sincere condolence to the bereaved family and sympathizes with them in their great grief.

### JOURNAL'S OWN PAGE.

Readers of the JOURNAL are invited to write; send us any item of interest that occurs in your practice; clinical notes of cases, etc., or, try your hand, if you have never written before, on a paper on some medical topic. Every physician of intelligence can relate in conversation what he sees and can verbally express, usually, good ideas on medical matters. Try putting your ideas on paper. You have only to put down in black and white what you would say if called on to discuss any given subject. (This is for beginners.) You need not be afraid of criticism. Tell those who would criticise you to try it themselves. Every writer—even those of fame—had to begin. The JOURNAL wants to encourage its readers to keep a record of important cases and to contribute the record to the literature on the subject. And—we want a variety. Send us your papers; they will be properly edited and put in shape for publication. All over the State societies are being formed and the members are contributing papers. Send them to the JOURNAL and let us put them in form for preservation.

### THE JOURNAL'S EXCHANGE BUREAU.

LOCATIONS AVAILABLE (IN TEXAS).

We have the following for sale. Our terms are 5 per cent., to be paid by the seller.

No. 1. A cash practice of $3000 to $4000 in a thickly populated Bohemian settlement—a nice town. Necessary to speak German to succeed the present incumbent. Can be secured by an investment of $500.

No. 2. A good practice in a flourishing stock and farm country, not far from Fort Worth; timbered country; the doctor has to go horseback. The purchase of a $500 residence secures the location—a good one.

No. 3. A good practice in Kerr county can be had by buying residence, store building, small stock of drugs; town of 500. Railroad, churches, schools, etc.; or party will exchange. Price not given.

No. 5. In Runnels county. Big practice can be secured. Incumbent has $3800 worth of property for sale, all or part. This is a very desirable location.

No. 6. In Tarrant county. 2 acres of land and a good two-story frame house; good soft water; every convenience of a home. Practice, $1600 a year average, collected for three years. $850, half cash; small town; nearest competition ten miles.

No. 7. In Walker county. Small railroad town; a farm of 52 acres,—35 in cultivation; 4-room house; rented to good tenant. Residence: house of 6 rooms, on a 22-acre tract, 8 in cultivation. Healthy location; nice, pleasant place, and desirable neighborhood, with all conveniences, such as church, school, etc. $1000 will buy the property and secure the practice. "The best bargain ever offered."

No. 8. Property in Florida. Orange orchards, etc., for sale or exchange for Texas property. Name given on request.

No. 9. A $2000 practice in a village in Milam county can be secured by buying a $500 residence. Purchaser inducted into the practice.

No. 10. A splendid opening for a physician can be had by purchasing a retiring doctor's home. It consists of a seven-room residence and lot; cost $3500; can be had for $2500, and the doctor will introduce the purchaser to his clientelle, remaining long enough with him to deliver the practice. Practice worth $3000. This is in one of the wealthiest and best sections in Texas, at the capital of one of the best counties.

No. 11. Practice established twenty odd years in a railroad town of about five hundred inhabitants, in one of the best counties in the State; six or seven acres, but improved property in town. Will sell or exchange for property in coast plains country.

In each instance satisfactory reasons for selling will be given. Parties who desire to purchase or exchange will please write us for name and address of advertisers, and mention the location desired, by number. Address inquiries to the.

TEXAS MEDICAL JOURNAL,
(Bureau of Exchange) Austin, Texas.

☞ Do not address any of the parties on a postal card, for obvious reasons.

